# FluCell-SELEX Aptamers as Specific Binding Molecules for Diagnostics of the Health Relevant Gut Bacterium *Akkermansia muciniphila*

**DOI:** 10.3390/ijms221910425

**Published:** 2021-09-27

**Authors:** Heinz Fabian Raber, Dennis Horst Kubiczek, Nicholas Bodenberger, Ann-Kathrin Kissmann, Deena D’souza, Hu Xing, Daniel Mayer, Pengfei Xu, Uwe Knippschild, Barbara Spellerberg, Tanja Weil, Frank Rosenau

**Affiliations:** 1 Institute for Pharmaceutical Biotechnology, Ulm University, Albert-Einstein-Allee 11, 89081 Ulm, Germany; Heinz.Raber@uni-ulm.de (H.F.R.); dennis.kubiczek@gmx.de (D.H.K.); nicholasbodenberger@gmx.de (N.B.); ann-kathrin.kissmann@uni-ulm.de (A.-K.K.); deena.dsouza@uni-ulm.de (D.D.); dolphinzyt2418@gmail.com (H.X.); 2 Max Planck Institute for Polymer Research Mainz, Ackermannweg 10, 55128 Mainz, Germany; weil@mpip-mainz.mpg.de; 3 Institute for Medical Microbiology and Hygiene, University Hospital Ulm, 89081 Ulm, Germany; Daniel.mayer@uniklinik-ulm.de (D.M.); barbara.spellerberg@uniklinik-ulm.de (B.S.); 4 Department of General and Visceral Surgery, Surgery Center, Ulm University, Albert-Einstein-Allee 23, 89081 Ulm, Germany; pengfei.xu@uniklinik-ulm.de (P.X.); uwe.knippschild@uniklinik-ulm.de (U.K.)

**Keywords:** aptamers, biosensors, FluCell-SELEX

## Abstract

Based on their unique properties, oligonucleotide aptamers have been named a gift of biological chemistry to life science. We report the development of DNA aptamers as the first high-affinity binding molecules available for fast and rapid labeling of the human gut bacterium *Akkermansia muciniphila* with a certain impact on Alzheimer´s disease. Fast and reliable analyses of the composition of microbiomes is an emerging field in microbiology. We describe the molecular evolution and biochemical characterization of a specific aptamer library by a FluCell-SELEX and the characterization of specific molecules from the library by bioinformatics. The aptamer AKK13.1 exerted universal applicability in different analysis techniques in modern microbiology, including fluorimetry, confocal laser scanning microscopy and flow cytometry. It was also functional as a specific binding entity hybridized to anchor primers chemically coupled via acrydite-modification to the surface of a polyacrylamide-hydrogel, which can be prototypically used for the construction of affinity surfaces in sensor chips. Together, the performance and methodological flexibility of the aptamers presented here may open new routes not only to develop novel *Akkermansia*-specific assays for clinical microbiology and the analyses of human stool samples but may also be an excellent starting point for the construction of novel electronic biosensors.

## 1. Introduction

*Akkermansia muciniphila* is a Gram-negative, oval shaped, strictly anaerobic bacterium with an ability to degrade mucin residing in the gut of mammals, including humans [1]. In healthy individuals, the composition of the microbial community of the intestinal tract—the gut microbiota—remains stable and resilient to ordinary physiological stresses such as alterations in nutrition or moderate variations of the corporal constitution. However, severe and significant disbalances in the count of individual bacteria in the intestinal flora have been recognized to account for a constantly increasing number of major serious diseases with a considerable impact on human society and health systems. The impact of *Akkermansia muciniphila* as one of the most prominent gut bacteria has been discussed in the context of different diseases including metabolic disorders such as obesity [2], diabetes mellitus [3], and neurodegenerative diseases such as multiple sclerosis [4,5], Alzheimer´s [6,7], and Parkinson´s disease [8]. In mouse models of Alzheimer´s disease, the development of symptoms is accompanied with a reduction of *A. muciniphila* in the gut microbiome [7]. Both the transfer of microbiota from healthy to sterile Alzheimer mice by stool transplantation as well as supplementation with *A. muciniphila* cells significantly reduced plaque formation in the brain [6,7]. Previous studies have delivered preliminary indication that this bacterium can be considered as safe for interventions in humans. When the abundance of *A. muciniphila* reached high levels of 60% in human gut after antibiotic treatment, no adverse effects were observed. In addition, in a clinical study, the safety and tolerability of *A. muciniphila* was evaluated in obese test persons [9]. The current state of *Akkermansia* research qualifies this fascinating bacterium as a promising future probiotic of general importance for the prevention and embankment of major scourges of human health.

With increasing evidence for the association of *A. muciniphila* with various clinical pictures, the necessity to analyze the gut microbiome composition and to monitor the success of probiotic supplementation becomes self-evident and requires powerful diagnostic tools. Current standards in microbiome analyses are techniques that involve nucleic acid sequencing or quantitative PCR for smaller subsets of bacteria. A general drawback of these methods is that they are laborious, time consuming and require specially trained personnel, severely limiting their suitability for routine check-ups during therapy [10]. Modern clinical microbiology therefore relies on diagnostic techniques, which are faster and, in their perfection, can even allow bedside diagnostics. This applies to fluorescence microscopy as a variant of a technique, which has been optimized since Paul Ehrlich´s times in the 19th century [11]. A current state of the art variant, confocal laser scanning microscopy (CLSM), which is a US Food and Drug Administration–approved optical imaging technology, can, for example, offer noninvasive visualization of skin lesions in vivo at nearly histologic resolution after appropriate staining [12].

Flow cytometry as a prototypically fast and precise analytical method allows accurate analyses on the single cell level and can thus be regarded as the irreplaceable work horse technique also in clinical diagnostics [13,14,15]. Biosensors have been introduced as the potential next generation of modern bioassays since they address secondary critical aspects of routine assay performance such as speed of detection, costs and specificity. The first biosensor was developed by Leland Clark in 1962 [16] and this enzyme-based glucose detection system was later introduced into the market in 1975 [17]. Since then, the biosensor field has developed exponentially, with more than 6000 publications in 2019 and has created various classes of biosensor principles. Most biosensors use specific biochemical reactions mediated by affinity molecules or isolated enzymes to detect chemical compounds by electrical, thermal, gravimetric or optical signals [18,19,20,21,22,23,24]. Antibodies as the predominant class of affinity molecules are currently continuously replaced by aptamers, which have been regarded as a highly valuable gift of chemical biology to science [25,26]. Unlike most ligands evolved in nature, these synthetic single stranded nucleic acid oligonucleotides with complex 3D structures are usually tolerant of harsh chemical, physical, and biological conditions [27]. These features qualify aptamers as potent and attractive molecular bioreceptors for the development of sensing devices [27]. In a typical iterative systematic evolution of ligands by exponential enrichment (SELEX) process, individual aptamers against a desired target are evolved from a library of 10^12^ to 10^16^ randomized individual sequences [28] by repetitive rounds of binding to the immobilized molecular target and removal of unbound aptamers. Recently, we developed the Fluorescence Whole Cell-SELEX (‘FluCell-SELEX’), which is based on the use of fluorescently labeled aptamers throughout the complete selection process and target structures naturally immobilized on surfaces of whole bacterial cells [26].

**Figure 1:**
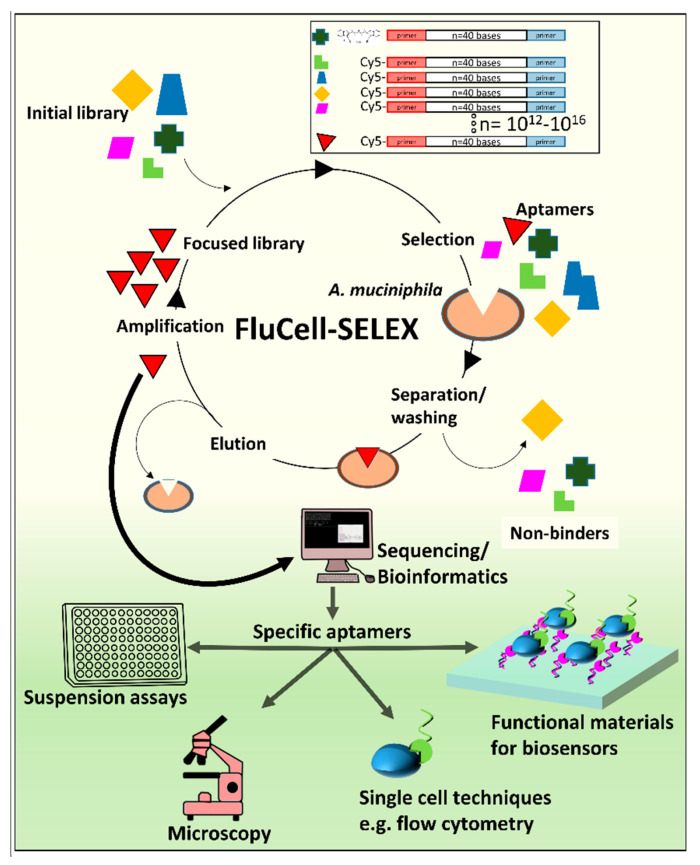
Selection of individual aptamers against *A. muciniphila* by the FluCell-SELEX [26] and their possible application in modern bioanalytical techniques. A randomized single stranded aptamer library was incubated with the target to separate binders from non-binders by simple washing and subsequent amplification of the bound aptamers to gain focused (i.e., specific) libraries. By repetition of this procedure with increasing stringency, a specific enriched polyclonal aptamer library was evolved and finally sequenced by next-generation sequencing to determine the most specific individual aptamer sequences by bioinformatics. The suitability of this aptamers in modern bioanalytical techniques including suspension assays, microscopy, flow cytometry and generation functional materials for biosensors was investigated.

Here, we describe the molecular evolution of an *Akkermansia muciniphila*-specific aptamer library by the FluCell-SELEX (Figure 1) and the selection of two individual aptamers with good specificities against *A. muciniphila* by next generation sequencing and bioinformatic analyses providing the numerical information by tracking the evolutionary trajectory of the individual sequences in response to the selection pressure during the SELEX. To the best of our knowledge, we have developed not only the first specific aptamers but also the first specific binding molecule in general for molecular recognition of this auspicious probiotic bacterium of emerging capital importance for human health. We have demonstrated that they are perfectly suited in fluorimetric microtiter plate assays, CLS microscopy and flow cytometry. Our results indicate that they are suitable for the functionalization of support materials without losing their specificity. Prospectively, this may pave the way for the development of innovative biosensors for the easy and cost effective detection of *A. muciniphila* and may inspire the scientific community and experts in the field of diagnostics to develop the next generation of assays deploying the aptamers introduced here.

## 2. Results

### 2.1. Evolution of the Focused Aptamer Library by FluCell-SELEX

**Figure 2:**
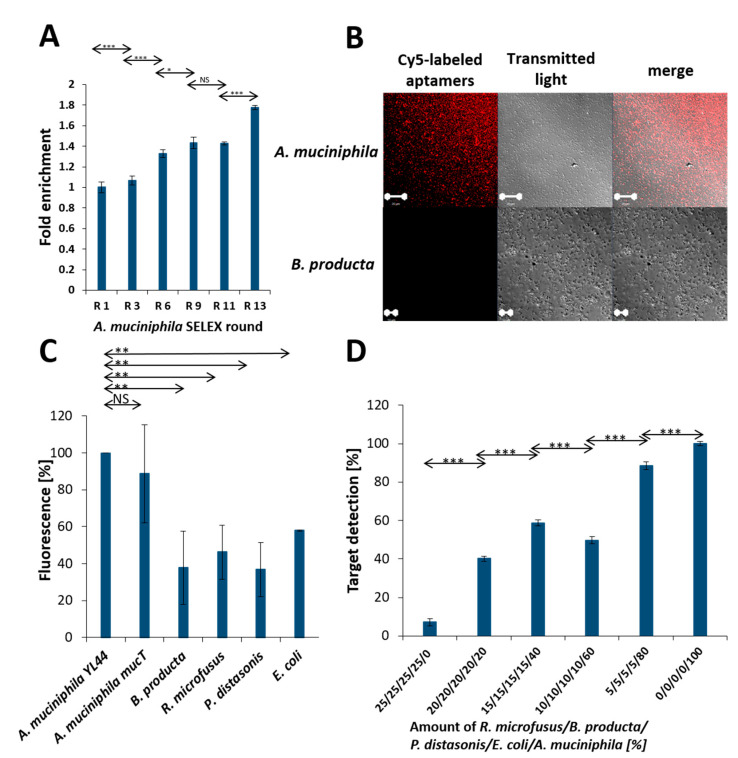
Binding and specificity of a polyclonal aptamer library selected against *A. muciniphila*. (**A**) Binding assay of fluorescently-labeled aptamer libraries at equal concentrations and washing with PBS. The fractions eluted from cells by heat show an increasing fluorescent signal, demonstrating the enrichment of specific aptamers. (**B**) Confocal laser scanning microscopy of aptamer labeled *A. muciniphila* cells. The fluorescently labeled polyclonal aptamer library was sufficient to label *Akkermansia* cells in situ. *Blautia producta* served as a control and did not display any colocalization of the aptamer with the cells. (**C**) Increased specificity of the polyclonal aptamer library toward *A. muciniphila* compared to the microbiome control organism. (**D**) The polyclonal library is sufficient to retrace the amount of *A. muciniphila* in a complex mixture including the gut microbiome control bacteria *B. producta*, *R. microfusus*, *P. distasonis* and *E. coli* were adjusted to equal optical densities and mixed at different volume ratios. Statistical analysis by a *t* test was performed for the enrichment of specific aptamers and the specificity of the polyclonal library. *p* values < 0.05 were considered significant. * denotes *p* < 0.05, ** *p* < 0.01 and *** *p* < 0.001. The experiments were performed in triplicate and standard deviations are given as error bars. When individually not visible, the deviation was too small to be marked by the graphic software.

The specificity of aptamers against *A. muciniphila* was evolved in a variant of the classical SELEX process based on the use of fluorescence labeling of the random library throughout the selection procedure. This so-called FluCell-SELEX [26] delivered the polyclonal library R13 after 13 rounds of selection under conditions, which increased the selection pressure by raising the stringency during the SELEX process and involved counter selections with *Blautia producta*, another prominent member of gut bacteria [29]. The efficiency of the aptamer based fluorescence labeling of *A. muciniphila* cells increased significantly in the individual SELEX rounds (Figure 2A). This cyanine 5 (Cy5)-labeled library allowed specific fluorescence labeling of *A. muciniphila* in confocal laser scanning microscopy (CLSM) and to definitely distinguish *A. muciniphila* from *B. producta* as a negative control strain (Figure 2B). In the microtiter plate based fluorimetric assay using the Cy5 labeled library [26] a series of experiments revealed that also in suspension two different *A. muciniphila* strains could reliably be differentiated from four control strains of gut bacteria with a high significance (Figure 2C). Moreover, this allowed retracing of increasing cell counts of *A. muciniphila* also in mixtures of these bacteria (Figure 2D).

### 2.2. Selection of Individual Aptamers from the Library

**Figure 3:**
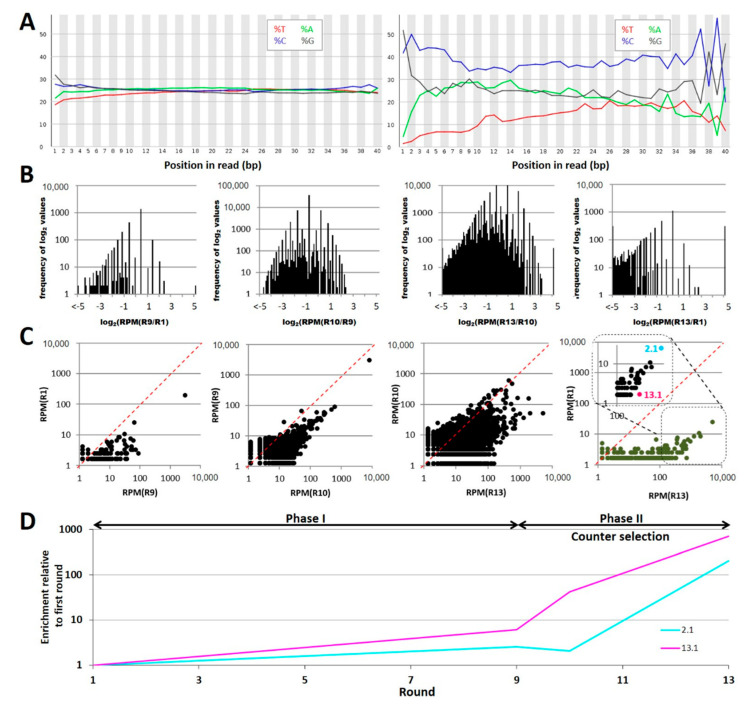
Bioinformatic analysis of the sequenced polyclonal aptamer library shows enrichment of individual aptamer sequences (**A**) Nucleotide distribution of the library R1 (left panel) and library R13 (right panel). The distribution of each nucleobase is given as a percentage; red lines indicate thymine (T), blue lines cytosine (C), green lines adenine (A), and grey lines cytosine (C). (**B**) Histogram of the relation of enrichment and depletion of individual sequences in different rounds. From left to right R1/R9, R9/R10, R10/R13, R1/R13. The histograms show significant depletion of individual sequences (log_2_ < 0) compared with progressing SELEX rounds. (**C**) Scatter plot comparing the sequences from rounds R1/R9, R9/R10, R10/R13, R1/R13 (left to right) shows an enrichment of individual sequences with increasing SELEX round numbers. The red lines represent an unchanged sequence distribution between the rounds. In the right scatter plot, the most enriched and the most abundant sequence were highlighted and chosen for further evaluation. (**D**) Enrichment profile of the sequences AKK13.1 and Akk2.1 during the SELEX process. Both Akk13.1 and Akk2.1 shows a slight enrichment in Phase I (increasing stringency of washing). In Phase II a counter-selection with *B. producta* was introduced resulting in a massive enrichment of AKK13.1 and Akk2.1.

Comparative next generation sequencing of the libraries resulted in 1.2 million reads for round 1, 1.6 million reads for round 9, 1.6 million reads for round 10, and 1.5 million reads for the final polyclonal library R13, respectively. Computational analysis of the relative nucleotide content within the randomized aptamer sequence regions of the aptamers proved a perfectly equal distribution in the first round of selection as an ideal starting situation for the enrichment of specific aptamers [30]. A shift toward higher GC contents was indicative for evolutional processes occurring during the SELEX resulting in drastically different sequences in the final library R13 (Figure 3A). Processing of the sequence populations with the “FASTAptamer compare toolkit” [31] resulted in the creation of 100 groups spanning binary logarithmic (log_2_) values of the frequency ratios from −5 to +5 in intervals of 0.1 and categorizes each group based on the number of calculated log_2_ values that result within each interval. Values that fall outside of this range populate two additional groups on either end of the distribution (<−5 or >5). The absence of any evolution between two libraries would mark one extreme representing Gaussian distribution narrowing log_2_ = 1. Shifts toward −5 or +5 indicate that individual sequences are depleted or enriched, respectively. Comparing rounds 1 with 9 and 9 with 10 major changes are represented by drastic depletion of sequences, an effect that is more pronounced in the comparison of 10 and 13 with log_2_ values ranging from −1 to <−5 (Figure 3B). Remarkably, also a sharp enrichment at log_2_ > 5 of only a few individual sequences occurred in addition and became utmost obvious in the R10/R13 comparison (Figure 3B, right panel). Scattered plotting of the frequencies of all sequences (as reads per million = RPM) being present in equal amounts in two sequence populations of the SELEX rounds to be compared results in a narrow distribution close to the diagonal. A shift of frequencies toward higher rounds was observed as a tendency representing a considerable enrichment of individual sequences, which again became markedly visible in the comparison of round 13 to 10 (Figure 3C). Aptamer Akk2.1 was the sequence with the highest abundance of all sequences in round 13 (marked in cyan, Figure 3C, right panel), while aptamer Akk13.1 showed the highest enrichment in the SELEX process (marked in magenta, Figure 3C, right panel). The SELEX was designed involving two distinct experimental phases, with phase I being characterized by an increase of stringency (round 1 to 9) and phase II involving counter selection against *B. producta*. The enrichment profiles of aptamer Akk2.1 and Akk13.1 reflect the stringency increase by likewise uniform enrichment, which is then further reinforced in phase II through the introduction of the counter selection (Figure 3D). As representatives of the two extremes (highest frequency and highest enrichment), both were selected for chemical synthesis and further analysis as the most promising candidates to identify specific *A. muciniphila* with a high affinity.

### 2.3. Application Testing

**Figure 4:**
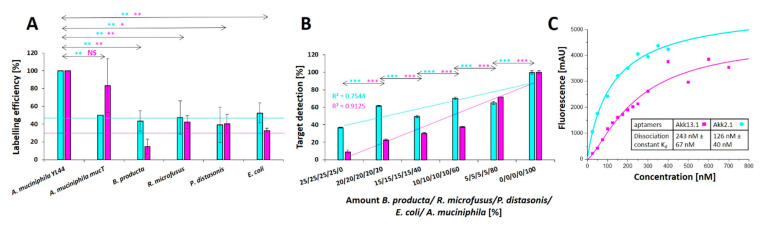
The aptamers Akk2.1 and Akk13.1 are highly potent in detecting *A. muciniphila* specifically. (**A**) Increased specificity of the aptamer Akk2.1 and Akk13.1 toward *A. muciniphila* compared to the microbiome control organism. The labeling background for Akk2.1 (cyan line) determined from the average fluorescence of the control organism is higher with 45% as the labeling background of Akk13.1 with 32% (magenta line) (**B**) Recovery of *A. muciniphila* from mixtures with microbiome associated organisms using the Akk2.1 and Akk13.1 aptamers. With an increasing amount of *A. muciniphila*, a higher fluorescent signal was detected, demonstrating the ability to detect of *Akkermansia* colocalized with other organisms. Statistical analysis by a *t* test was performed for the specificity of the individual aptamers and the detection of *A. muciniphila* in complex mixtures. *p* values < 0.05 were considered significant. * denotes *p* < 0.05, ** *p* < 0.01 and *** *p* < 0.001 (**C**) *A. muciniphila* incubated with different concentrations of the Akk2.1 and Akk13.1 aptamer to determine the dissociation constant. From the increasing fluorescent signal a dissociation constant of 126 nM for AKK2.1 and of 243 nM for AKK13.1 was determined.

Both Cy5-labeled aptamers Akk2.1 and Akk13.1 were suitable to differentiate *A. muciniphila* YL44 from single microbiome control strains with a lower labeling background of 32% for Akk13.1 instead of 45% for Akk2.1. However, Akk13.1 not only allowed labeling of the *A. muciniphila* YL44 originally isolated from mice but also the *A. muciniphila* strain mucT originating from human gut with high accuracy (Figure 4A). This was also the case when the *A. muciniphila* target cells were mixed with the individual control strains (Figure S1), as well as in experiments for the detection of *A. muciniphila* in complex mixtures of all the microbiome control strains, in which both aptamers allowed retracing of increasing amounts of their dedicated target cells (Figure 4B). However, for Akk2.1, the deviation of the coefficient of determination (R^2^) was 0.75 and thus indicative for a non-linear correlation of *A. muciniphila* cell densities in the mixtures. Compared to this, Akk13.1 detected *A. muciniphila* in the mixtures in a linear correlation of detection and cell count with a satisfactory coefficient of determination (R^2^ = 0.91). Moreover, spiking of the stool content of mouse cecum with increasing counts of additional *A. muciniphila* cells revealed that a surplus of 1–10% of the published count of *A. muciniphila* cells in mouse cecum [32] could be retraced in the samples (Figure S2). The dissociation constants of 126 (+/− 40) nM for the aptamer Akk2.1 and 243 (+/− 67) nM were in a reasonable range for accurate cell labeling applications (Figure 4C).

**Figure 5:**
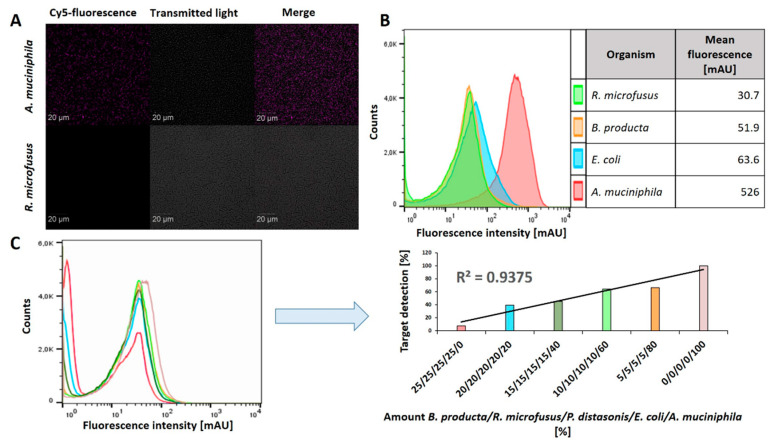
Akk13.1 is suitable for application in modern bioanalytical techniques. (**A**) Confocal laser scanning microscopy of aptamer labeled *A. muciniphila* cells. The fluorescent labeled aptamer Akk13.1 was sufficient to label *Akkermansia* cells. *R. microfusus* served as a control and did not display any colocalization of the aptamer with the cells. (**B**) Specific identification of *A. muciniphila* by flow cytometry analyses. *A. muciniphila*, *R. microfusus*, *A. stercoricanis*, *E. coli* were incubated with the aptamer and analyzed by flow cytometry measurements. The mean fluorescence of *A. muciniphila* is 8- to 17-fold higher in comparison to the other organism. (**C**) Identification of *A. muciniphila* contaminated with the microbiome associated organisms *B. producta*, *R. microfusus*, *P. distasonis* and *E. coli*. The fluorescence signal correlates with the increasing amount of *A. muciniphila* in the mixture.

State-of-the-art methods for microbiological analyses or diagnostic in clinical microbiology are single cell methods including microscopy (e.g., CLSM) and flow cytometry. The aptamer Akk13.1 delivered slightly more reliable results with the cecum samples (Figure S2), but—more important—the background labeling of single microbiome control bacteria was lower, and it outperformed the aptamer Akk2.1 in the retracing experiments with the minimalized model gut consortia. In summary, this qualified the *A. muciniphila* specific aptamer Akk13.1 to be evaluated for its potential prospective implementation in *Akkermansia* adapted variants of the aforementioned analytic and diagnostic methodologies. Since the bacterium *R. microfusus* has the highest background in the microtiter based labeling experiments it served as the most demanding control from our set of bacteria. Nevertheless, it completely failed to be labeled in CLS microscopy, whereas *A. muciniphila* was perfectly stained by aptamer Akk13.1 (Figure 5A).

Flow cytometry with the individual microbiome bacteria proofed the higher precision of this single cell method compared to the fluorimetric microtiter plate assay because the *A. muciniphila* signal was one order of magnitude higher and its peak was distinctively remote from the peak ensemble of the control strains (Figure 5B). Analysis of the bacterial mixtures was also possible with a reliable linear correlation of the signal with the cell count (R^2^ = 0.94) (Figure 5C).

**Figure 6:**
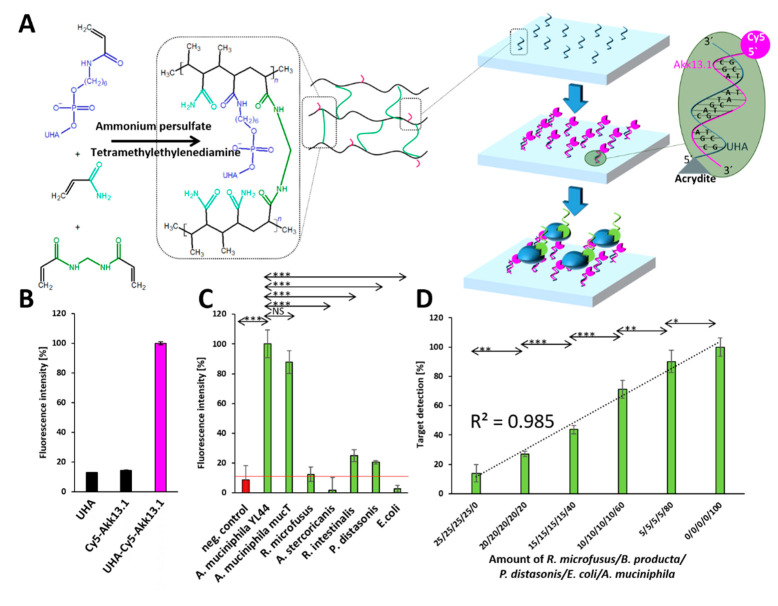
Design of an Akk13.1 based functional material for future biosensor applications (**A**) Reaction mechanism of the prototype aptamer-polyacrylamide based *A. muciniphila* detection system. The polyacrylamide-gel is modified by a universal hybridization anchor (UHA) consisting of an acrydite-modified single stranded DNA sequence complementary to the flanked regions of Akk13.1. The complementary primer binding sites of the Cy5-labeled aptamer Akk13.1 (Cy5-Akk13.1) are bound to the UHA and are able to catch specific *A. muciniphila* out of a sample. The second Oregon green 488 labeled “detection” aptamer Akk13.1 binds to the immobilized bacteria. (**B**) Binding control of the “catching” aptamer to the acrylamide modified primer polymerized to the hydrogel. There is a high amount of aptamer binding to the modified polyacrylamide-gel in comparison to unmodified polyacrylamide-gel (**C**) Specific detection of *A. muciniphila* with the Akk13.1 based functional material. The fluorescence signal of the suspension containing *Akkermansia muciniphila* is significant higher in comparison the signal from the gut microbiome control organism. (**D**) *A. muciniphila* can be retraced in mixtures with the microbiome associated organisms *B. producta*, *R. microfusus*, *P. distasonis* and *E. coli*. The mixtures contributed of varying amounts of *R. microfusus/B. producta/P. distasonis/E. coli/A. muciniphila*. The fluorescence signal correlates with the increasing amount of *A. muciniphila*. *t*-Test was performed where indicated with *p* values < 0.05 were considered significant. * denotes *p* < 0.05, ** *p* < 0.01 and *** *p* < 0.001.

Implementation in biosensor devices requires affinity molecules to be immobilized on dedicated support materials such as polyacrylamide gels, which have been used since 1970 [33] and can be functionalized with single stranded DNA [34]. Antibody based immunosorbent assays typically involve different antibodies as affinity molecules to immobilize the analyte (“capture” antibody) and a second antibody for the subsequent detection of the analyte on the surface (“detection” antibody). Here, this principle was modified by replacing the antibodies with aptamers for capture and detection. The solid phase was based on a polyacrylamide gel presenting a functional layer exposing Akk13.1 for capturing *A. muciniphila* cells as the targets. Acrydite functionalized universal anchor (UHA) oligonucleotides providing complementary sequences for immobilizing the capture aptamer to the solid phase by hybridization were incorporated in the radical polymerization of bisacrylamide and acrylamide molecules. After binding of the target cells to this functionalized surface, they are detected by a secondary aptamer (“detection” aptamer) labeled with the fluorescent dye, in this case, Oregon green 488 coupled to the 5′ end of the Akk13.1 (Figure 6A). As a capture aptamer, Cy5 labeled Akk13.1 was used while the same aptamer served as the detection aptamer as an Oregon green 488 labeled derivative. As expected, the capture aptamer was only present on the material surface when the UHA oligonucleotide had been incorporated during the fabrication process. In contrast, without UHA, no noteworthy (unspecific) binding of the capture aptamer to the solid phase was observed (Figure 6B). Furthermore, if the solid phase was not functionalized by the capture aptamer, the bacteria were not immobilized, which resulted in the missing signal upon the administration of the detection aptamer. However, the complete system consisting of the immobilized capture aptamer, which binds *A. muciniphila* specifically and the detection aptamer for fluorescent target labeling was able to distinguish *A. muciniphila* from the control strains (Figure 6C). In addition, in complex mixtures, the amount of *A. muciniphila* could be perfectly retraced quantitatively with a coefficient of determination (R^2^) of 0.985 (Figure 6D).

## 3. Discussion

Modern diagnostic methods in clinical microbiology include fluorescence based variants of microscopy, flow cytometry or functional materials for the detection of target cells in biosensors. These techniques in general require appropriate (labeled) affinity molecules for target binding, which have traditionally been antibodies, but that will be replaced by specific aptamers in the next decade. For the first time, we have used the FluCell-SELEX to evolve two specific aptamers against *A. muciniphila* as one of the most important health related gut bacterium with a yet finally unassigned high potential as a beneficial probioticum for humans. Our study demonstrates that these anti-*Akkermansia* aptamers perfectly meet the technical demands of affinity molecules for CLS microscopy, flow cytometry and as affinity entities on polyacrylamide gels as a simple model carrier material for the construction of solid phases for biosensors in the future.

Aptamers as a relatively new class of binding molecules are entering the field of diagnostics with an impressive pace; however, with the target are cells most of the studies address cancer-related issues, whereas microbiological targets are underrepresented, and in most cases, are focused on prominent pathogenic bacteria. Nevertheless, commercial aptamers are entering clinical diagnostics as true applications to predict the efficacy of immune therapies against breast or prostate cancer [35]. In contrast, promising aptamers evolved against human and animal pathogenic bacteria such as *Pseudomonas aeruginosa* and *Staphylococcus aureus* or against germs such as *Salmonella spec.* and *Listeria spec.* responsible for foodborne diseases are still awaiting their advancement into diagnostic products and their commercial introduction to the market [26,36,37,38]. The development of aptamer assays for the analysis of the composition of gut microbiomes or for monitoring the success of probiotic supplementation treatments in the context of various diseases is currently culpably disregarded, although this may represent a veritably new and promising branch in clinical microbiology of unneglectable potential for commercial success in the near future.

*A. muciniphila* probably represents the next level of probiotic strains because it has been discussed in the context of severe diseases of the modern industrial community with strongly aging populations such as obesity or neurodegenerative disorders including Alzheimer´s disease [7]. Recent studies demonstrate that supplementation with *Akkermansia* can be considered as safe in humans and suggest a beneficial or therapeutic effect on the course of those diseases [2,9]. Thus, it may be one of the most attractive chances in current clinical microbiology to develop fast, reliable, and if possible, a cheap and easy way to handle aptamer assays for monitoring the success of *Akkermansia* supplementation and the resulting alterations of microbiome composition in stool samples. Recently, we have shown that reliable molecular diagnostic of the human pathogen *P. aeruginosa* can be conducted simply by using a focused polyclonal aptamer library, as it results from the SELEX process without the need to identify and characterize individual sequences to generate defined single affinity molecules. However, this concept to use a polyclonal library was shown to require sophisticated biotechnological production strategies, allowing the long-term maintenance of the diversity (i.e., the available functional sequence space) in the library composition [26,35,39,40]. The polyclonal library after 13 rounds of selection against *A. muciniphila* was similarly well suited and allowed to specifically label and distinguish *Akkermansia* cells from other gut bacterial, which served as controls in a minimal model gut microbiome in our experiments. Nevertheless, we decided to follow the classical textbook-like strategy by extracting individual best performer sequences because defined single aptamers can be easily produced by solid phase synthesis, which guarantees a higher reproducibility compared to enzymatic amplification of the polyclonal library. This is also supportive for our main intention to provide *Akkermansia*-specific sequences as binding entities to the scientific community to inspire experts in the field of assay development to construct new diagnostic biosensor platforms hereon.

The success of the FluCell-SELEX procedure was virtually ideal and taken from a textbook [26,41], and thus delivered the best binding aptamers Akk2.1 and Akk13.1 as exposed by their obvious enrichment profiles. These DNA oligonucleotides were then produced by phosphoramidite solid phase synthesis as two different variants 5′-labeled either with cyanine 5 or Oregon green 488, a fluorinated derivative of fluorescein, and both dyes did not affect the functionality nor their three-dimensional structure. The performance of the aptamers was evaluated by suspension fluorimetric labeling assays, flow cytometry and confocal microscopy, methods that represent state-of-the-art diagnostic techniques. Criteria for a good performance were i) the possibility to unequivocally distinguish *Akkermansia* from the control strains with a low labeling background, ii) to retrace different amounts of *Akkermansia* in mixtures of bacteria, and iii) a linear correlation to these “contaminating” cells accompanied by coefficients of determination (R^2^) of at least 0.9. Although both Akk2.1 and Akk13.1 in principle met these criteria, Akk13.1 clearly outperformed Akk2.1 with a low labeling background of 35% and an R^2^ value of 0.91 compared to 45% and R^2^ = 0.75 for Akk2.1 in the fluorimetric microtiter plate suspension assays. Due to this, we decided to select Akk13.1 and to further evaluate only this most promising affinity molecule. Akk13.1 was perfectly suited in CLS microscopy and allowed specific labeling of *Akkermansia* and to distinctively discriminate it from controls. Furthermore, in flow cytometry, Akk13.1 brought a great labeling performance of *Akkermansia* with a slight labeling background of 9% for the controls and was greatly capable of retracing it with an R^2^ of 0.94 from complex mixtures. The initial retracing experiments with samples from mice intestines spiked with defined amounts of *A. muciniphila* from classical laboratory cultures are far from being comprehensive but suggest a considerable ability of the aptamer Akk13.1 to specifically bind to *Akkermansia* cells, allowing quantitative labeling in bacterial mixtures of high complexity. The construction of aptamer based biosensors requires the quantitative immobilization of aptamers on technical surfaces with gentle techniques, allowing to maintain the productive and functional 3D structure of these affinity entities. In addition to classical crosslinking reactions for the functionalization of materials with nucleic acids, an elegant strategy was introduced by Li and coworkers in 2015, which uses acrydite-modified anchoring oligonucleotides copolymerized in a polyacrylamide gel and complementary sequences at one end of the dedicated binding aptamer flanking the functional actual aptamer sequence, thus allowing its immobilization to the gel surface by hybridizing to the anchor primer. In our concept, the UHA oligonucleotides were copolymerized to the material via their 5′ acrydite modification to the material and provided the hybridizing sequences, which were complementary to the 3′ primer binding regions of Akk13.1. The binding of Akk13.1 to the solid phase exclusively depended on the presence of UHA, and in this immobilized form, the aptamer retained its functionality on the resulting material. Maintaining full functionality on surfaces is supportive for our opinion that Akk13.1 represents a promising affinity molecule as the fundament for the development of state-of-the-art types of biosensors (e.g., electronic or optical) which however, still requires a considerable amount of effort by experts from the biosensor field. This is especially the case for the construction of dedicated biosensor devices, which should allow clinically validated quantitative analyses of human samples such as stool samples of patients.

## 4. Materials and Methods

### 4.1. Cultivation of Bacteria

The bacteria strains *A. muciniphila* YL44 (DSMZ-26127), *A. muciniphila* mucT (DSMZ-22959) [1,42], *Escherichia coli* DH5α (DSMZ-6897), *Blautia producta* (DSMZ-14466) [29], *Rikenella microfusus* (DSMZ-15922) [43,44], *Allobaculum stercoricanis* (DSMZ-13633), Roseburia intestinalis (DSMZ-14610) and *Parabacteroides* distasonis (DSMZ-29491) [45,46] were cultivated in Oxoid^TM^ anaerobe basal broth (Oxoid Ltd., Basingstoke, UK) Medium at 37 °C under anaerobic conditions (90% Nitrogen/10% Hydrogen).

### 4.2. SELEX Procedure

SELEX was performed by a FluCell-SELEX as described previously [26,47]. Briefly, SELEX was performed in two phases. After an initial selection round using 1 nmol of a randomized aptamer library (TriLink Biotechnologies, San Diego, CA, USA) in phosphate buffered saline (PBS; Thermo Fisher Scientific, Waltham, MA, USA), the aptamer concentration was kept constant at 60 nM in all selection rounds. In phase 1, the aptamer solution contained tRNA (Thermo Fisher Scientific, Waltham, MA, USA) and bovine serum albumin (Carl Roth, Karlsruhe, Germany) with concentration of 1.2 µM in selection round 1 increasing in 600 nM steps until round 8 to 5.4 µM. Additionally, washing steps with 1 mL PBS were performed once in round 1 and 2, 3 times in round 3 and 4, four times in round 5, 5 times in round 6, and 7 and 6 times from round 8 and onward. In phase II, a counter-selection with the gut control strain *Blautia producta* was introduced in round 10. Therefore, a 1 mL cell suspension with an OD_600nm_ of 1 was pre-incubated with the aptamer solution for 1 h before the aptamers were transferred to *A. muciniphila* for positive selection. To maximize the stringency from round 12, the PBS used for washing contained an additional 0.05% (*w*/*v*) BSA. Between the selection rounds, the eluted aptamers were amplified by PCR (Forward primer sequence: 5′-[Cy5]-TAGGGAAGAGAAGGACATATGAT-3′ and reverse primer sequence: (5′-Biotin-TCAAGTGGTCATGTACTAGTCAA-3′).

### 4.3. Suspension Fluorimetric Labeling Assay

During the SELEX procedure as well as for individual aptamers later on, binding assays were performed in a suspension fluorimetric labeling assay. Therefore, aptamers fluorescently labeled with Cyanine 5 (excitation: 635 nm, emission: 670 nm) at their 5′ end were used. Testing of specificity toward individual organisms as well the binding in bacterial mixtures was performed using a 60 nM aptamer solution. Bacterial cultures were adjusted to an OD_600nm_ of 1 and mixed in distinct volume ratios for bacterial mixtures. A volume of 1 mL of bacterial suspension was centrifuged for 2 min at 11,000 rpm and washed with 1 mL PBS. The pellet was resuspended in 500 µL of the previously prepared, activated aptamer solution. In the mice intestine content experiment, 10 mg of cecal content of mice was added to the solution additional. After incubation for 30 min, cells were centrifuged for 2 min at 3000 rpm and the supernatant was discarded. After washing with 500 µL PBS, cells were centrifuged respectively, and the pellet was resuspended in 100 µL PBS. Elution of aptamers was achieved by heating to 95 °C for 5 min and subsequent centrifugation at 11,000 rpm. The fluorescence of the eluted aptamers was measured in the supernatant with an excitation wavelength of 635 nm and an emission wavelength of 670 nm using a Tecan infinite M200 microplate reader (Tecan group AG, Männedorf, Switzerland). For binding constant determination, the assay was performed equally using different aptamer concentrations. The binding constant was then derived from a one site specific binding plot according to the equation $\;F=\frac{F_{max}\cdot {\left[c\right]}^{n}}{K_{d}+{\left[c\right]}^{n}}$, with *F* = the measured fluorescence, *F_max_* = the maximal fluorescence, [c] = concentration of aptamer, *n* = hill coefficient and K_d_ = the binding constant.

### 4.4. Next Generation Sequencing

After 13 rounds of selection, aptamers were extended with the primers 5′-ACGATGATACTCGGACTGTAGGGAAGAGAAGGACATATGAT-3′ and the reverse primer 5′-TCTCGTAGTTCAAGC GACTCAAGTGGTCATGTACTAGTCAA-3′ to introduce universal primer binding sides (underlined) and to allow bridge formation necessary for Illumina sequencing^®^ (GATC Biotech AG, Konstanz, Germany). The PCR was performed using a Phusion^®^ DNA polymerase (NEB Inc., Ipswich, MA, USA) according to the manufacturers protocol in a three-step thermal reaction: initial denaturation for 3 min at 94 °C, 9 cycles of 94 °C for 30 s, 49.1 °C for 30 s and 72 °C for 30 s, and a final extension for 2 min at 72 °C. In a second step using the purified extended aptamers (QIAquick PCR purification kit, Qiagen, Venlo, The Netherlands) as template, index sequences were introduced according to the recommendations of Tolle and Mayer [47]. This was performed for aptamers recovered after 1, 9, 10 and 13 rounds of selection using individual primers for index introduction (Table 1). The PCR was performed analog to the first extension with a modified annealing temperature of 56.6 °C.

Intervention studies involving animals or humans and other studies that require ethical approval must list the authority that provided approval and the corresponding ethical approval code.

### 4.5. Computational Analysis of NGS Data

NGS quality was checked with the FastQC toolkit [48] and showed an average Phred score >35 within the aptamer region. Further preprocessing, including the sorting of the sequences based on the introduced indices as well as the clipping of the primer binding sites and analysis of the nucleotide distribution, was performed by using the FastQC and FASTX toolkit [49]. Sequences were collapsed, sorted and analyzed regarding their enrichment by the FASTAptamer toolbox [31]. Finally, the secondary structure of the chosen candidates was then predicted by using the Mfold server [50] for a folding temperature of 25 °C and a Na^+^ concentration of 137 mM. Sequences of Akk2.1: GGAAGAGAAGGACATATGATGCGGGGA GAGGCGAAAGAAGCTGGGATGGAAGGGCGTAGGTTGACTAGTACATGACCACT and Akk13.1: GGAAGAGA AGGACATATGATCCGCCACACCACCACAGCTCGCCGACCGACTCACCGCGCCTTG ACTAGTACATGACCACT.

### 4.6. Evaluation of Akk13.1 in Flow Cytometry

Aptamer binding was performed as described above. After washing with 500 µL PBS, the cells were resuspended in 500 µL PBS, and labeled cells were measured using a BD FACSCalibur (BD Bioscience, Franklin Lakes, NJ, USA) and excitation of 630 nm and an emission detection of 670 nm. The mean fluorescence of 250,000 counted cells was determined by the software FlowJo (BD Bioscience, Franklin Lakes, NJ, USA)

### 4.7. Evaluation of Akk13.1 in Microscopy

Aptamer labeling was performed as described above. After washing with 500 µL PBS, the cells were resuspended in 500 µL PBS and analyzed by an inverted Carl Zeiss 710 confocal laser scanning microscope (Carl Zeiss, Oberkochen, Germany) with an excitation wavelength of 633 nm.

### 4.8. Evaluation of Akk13.1 for Biosensor Applications

The solid phase was generated by polymerization of acrylamide/bisacrylamide (29:1) solution (30 µL, 10% *w*/*v*, Carl Roth, Karlsruhe, Germany) and the acrydite-modified UHA (2.7 µM, sequence: 5’-[acrydite]-AGGGAAGAGAAGGACATATGAT-3′, IDT, Iowa, USA) with ammonium persulfate (APS, 3 µL, 10% *w*/*v*, Carl Roth, Karlsruhe, Germany) and N,N,N’,N’-tetramethylenediamine (TEMED, 3 µL, 10% *v*/*v*, Carl Roth, Karlsruhe, Germany) in a 96-well plate (Sarstedt, Nümbrecht, Germany). The modified solid phase was incubated with a 100 µL Cy5-labeled Akk13.1 capture aptamer solution in PBS for 4.5 h and washed 3 times with 100 µL PBS to remove the unbound aptamers.

Bacterial cultures were adjusted to an OD_600nm_ of 1 and mixed in distinct volume ratios for bacterial mixtures. A volume of 200 µL of bacterial suspension was centrifuged for 2 min at 11,000 rpm and washed with 200 µL PBS. The pellet was resuspended in 200 µL PBS and incubated on the solid phases for 1 h. After washing with 100 µL PBS, the solid phase was incubated with a 100 µL Oregon green 488 labeled Akk13.1 detection aptamer solution in PBS (60 nM) for 1 h and washed with 100 µL PBS. The fluorescence of the solid phase was measured with a Tecan infinite M200 microplate reader (Tecan group AG, Männedorf, Switzerland) at 485 nm excitation wavelength and 535 nm emission wavelength.

### 4.9. Animal Housing and Breeding

Sixteen-week-old male and female C57BL/6J mice were purchased from the in-house breeding facility of Ulm University. They were kept in a pathogen-free open cage facility in a 12 h light/dark cycle at 22.5 ± 1 °C. The mice had access to food and water ad libitum and were used for breeding purposes until exhausted by law. All animal experiments were approved by state and local authorities (Regierungspräsidium Tübingen, Ulm University/license number: 1183). Furthermore, the animal experiments were conducted in accordance with ARRIVE guidelines and local regulations. The twenty-week-old mice were sacrificed by CO_2_ inhalation and the colon was isolated, snap frozen in liquid nitrogen, and stored at −80 °C until further use.

## 5. Conclusions

In summary, we provide the first aptamer library and selected from this library the first individual DNA aptamer as a high-affinity binding molecule with specificity against *A. muciniphila*. We described the possibility of using these aptamers in different diagnostic methodologies and demonstrated that the specific aptamer Akk13.1 maintains its binding functionality toward this important Gram-negative member of the human gut microbiome, including after its immobilization on synthetic surfaces. We believe that this may pave new avenues toward fast and reliable diagnostics of *A. muciniphila* in real clinical human samples, including in a new generation of biosensors in the future.

## Figures and Tables

**Table 1:** Primers used for index introduction (index sequences are illustrated in bold).

Round	Index 5′ → 3′	Forward Primer 5′ → 3′	Reverse Primer 5′ → 3′
1	ATC ACG	TCAGTCGTAT **ATCACG** ACGATGATACTC GGACTGTAGGGAAGAGAAGGACATATGAT	GCTATGTACT **CGTGAT** TCTCGTAGTTCA AGCGACTCAAGTGGTCATGTACTAGTCAA
9	CGA TGT	TCACTCGTAT **CGATGT** ACGATGATACTC GGACTGTAGGGAAGAGAAGGACATATGAT	GCTATGTACT **ACATCG** TCTCGTAGTTCA AGCGACTCAAGTGGTCATGTACTAGTCAA
10	TTA GGC	TCACTCGTAT **TTAGGC** ACGATGATACTC GGACTGTAGGGAAGAGAAGGACATATGAT	GCTATGTACT **GCCTAA** TCTCGTAGTTCA AGCGACTCAAGTGGTCATGTACTAGTCAA
13	TGA CCA	TCACTCGTAT **TGAACCA** ACGATGATACTC GGACTGTAGGGAAGAGAAGGACATATGAT	GCTATGTACT **TGGTCA** TCTCGTAGTTCA AGCGACTCAAGTGGTCATGTACTAGTCAA

## Supplementary Materials

The following are available online at https://www.mdpi.com/article/10.3390/ijms221910425/s1.
