# Supplementary material for: FluCell-SELEX Aptamers as Specific Binding Molecules for Diagnostics of the Health Relevant Gut Bacterium Akkermansia muciniphila
Source: Int J Mol Sci. 2021 Sep 27;22(19):10425. doi: 10.3390/ijms221910425 (PMC8509069; doi:10.3390/ijms221910425)
Supplement: Supplementary file 1 [file ijms-22-10425-s001.zip › ijms-1380442-supplementary.pdf]

Supplementary

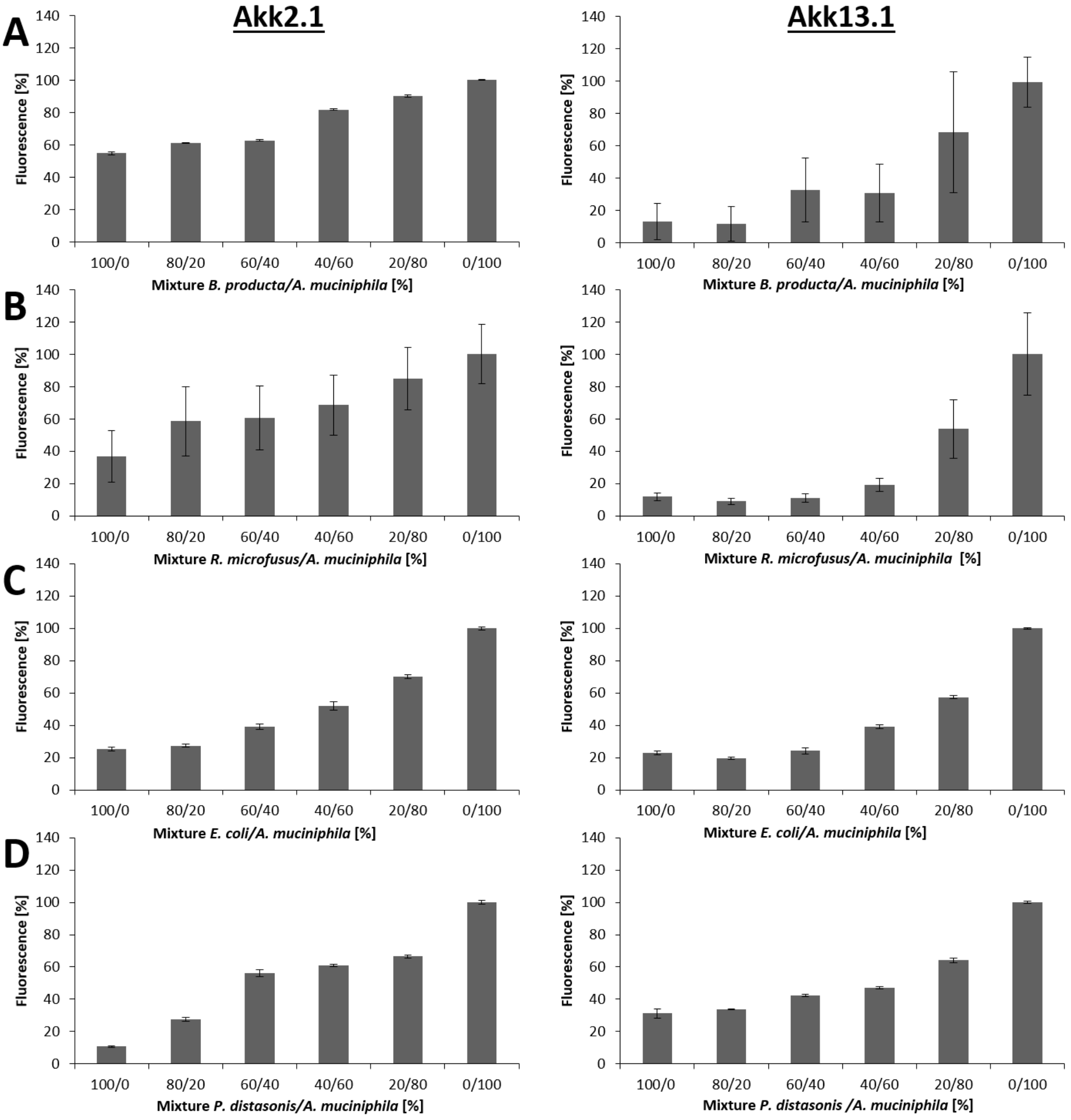

Supplementary Figure S1: Recovery of *A. muciniphila* from mixtures with microbiome associated organisms using the Akk2.1 and Akk13.1 aptamers. Cells were adjusted to equal optical densities and incubated with the fluorescently labelled aptamer Akk2.1. or Akk13.1. With increasing portions of *A. muciniphila* a higher fluorescent signal was detected in the eluted fraction demonstrating the detection of *Akkermansia* colocalized with other organisms. (a) Mixture consisting of *B. producta* and *A. muciniphila* detected with the aptamer Akk2.1. (b) Mixture consisting of *B. producta* and *A. muciniphila* detected with the aptamer Akk13.1. (c) Mixture consisting of *R. microfusus* and *A. muciniphila* detected with the aptamer Akk2.1. (d) Mixture consisting of *R. microfusus* and *A. muciniphila* detected with the aptamer Akk13.1. (e) Mixture consisting of *E. coli* and *A. muciniphila* detected with the aptamer Akk2.1. (f) Mixture consisting of *E. coli* and *A. muciniphila* detected with the aptamer Akk13.1. (g) Mixture consisting of *P. distasonis* and *A. muciniphila* detected with the aptamer Akk2.1. (e) Mixture consisting of *P. distasonis* and *A. muciniphila* detected with the aptamer Akk13.1.

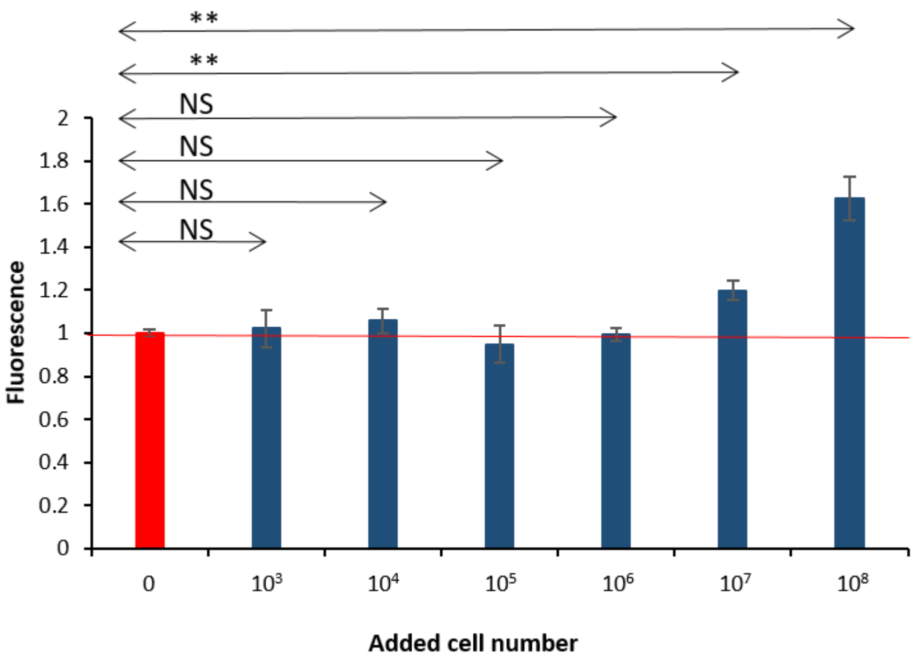

Supplementary Figure S2: Recovery of *A. muciniphila* from mixtures with a mice intestinal content solution using the aptamer Akk13.1. Cells were adjusted by their optical density to the desired cell number and incubated with 30 pmol fluorescently labelled aptamers and mice intestinal content. Increasing amounts of *A. muciniphila* cells ranging from 10^3 to 10^8 cells were added to the intestinal content, where ~10^8 *A. muciniphila* cells were already present. From an *Akkermansia* amount of O.D.$_{600}$ of 0.1an increasing fluorescence signal was detected from the eluate fraction demonstrating the detection of *Akkermansia* under realistic measurement conditions in diagnostic. Red line indicates the base signal gained from the intestinal content itself. P values < 0.05 were considered significant. ** denotes < 0.01, NS denotes not significant.

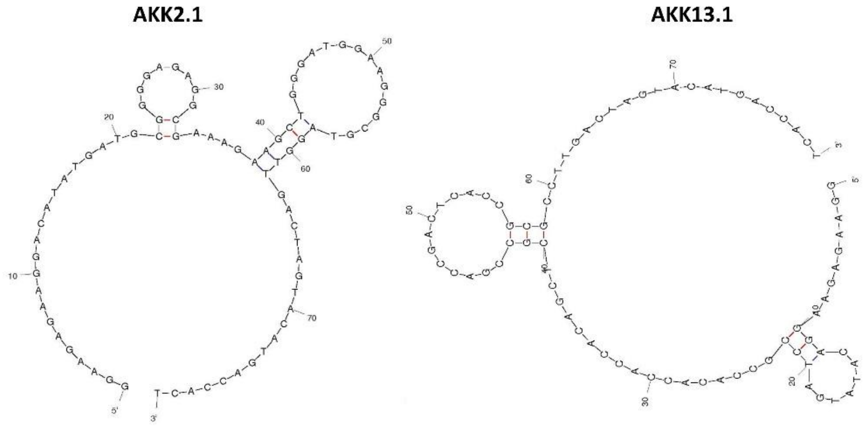

Supplementary Figure S3: 2D-structure of AKK2.1 and Akk13.1 as simulated with the mfold web tool[50]. The secondary structure of the chosen candidates was then predicted for a folding temperature of 25°C and a Na^+ concentration of 137 mM.Akk2.1: GGAAGAGAAGGACATATGATGCGGGGAGAGGCGAAAGAAGCTGGGATGGAAGGGCGTAGGTTGACTAGT ACATGACC ACT and Akk13.1: GGAAGAGAAGGACATATGATCCGCCACACCACCACAGCTCGCCGACCGACTCACCGCGCCTTGACTAGTAC ATGAC CACT.
